# UV/Visible Diffusion-Ordered Spectroscopy: A Simultaneous Probe of Molecular Size and Electronic Absorption

**DOI:** 10.1021/acs.analchem.4c02026

**Published:** 2024-09-10

**Authors:** Giulia Giubertoni, Marina Gomes Rachid, Carolyn Moll, Michiel Hilbers, Saer Samanipour, Sander Woutersen

**Affiliations:** Van ’t Hoff Institute for Molecular Sciences, University of Amsterdam, Science Park 904, Amsterdam 1098XH, The Netherlands

## Abstract

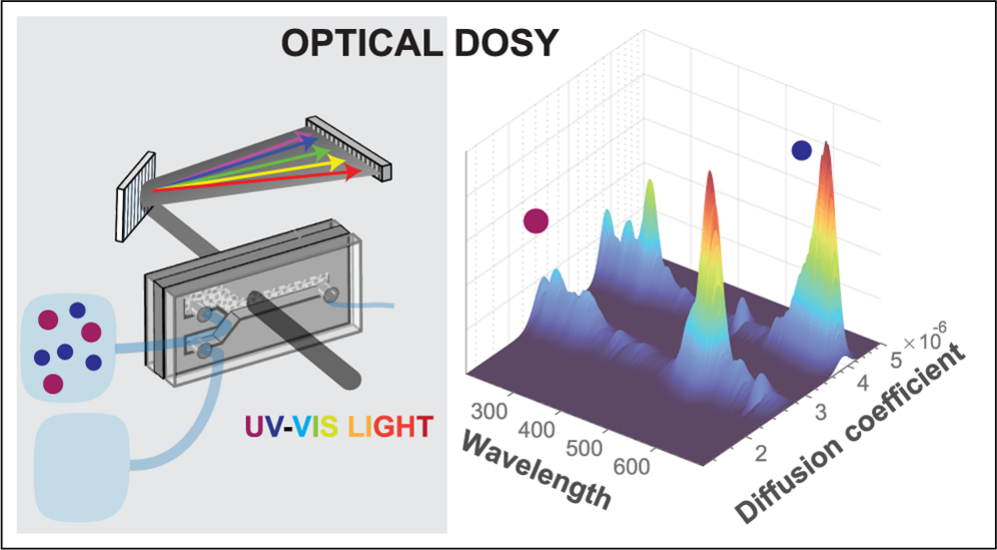

Based on concepts from nuclear magnetic resonance, we have developed UV/vis diffusion-ordered spectroscopy, which simultaneously probes the size and electronic absorption spectrum of molecules and particles. We use simple flow technology to create a step-function concentration profile inside an optical sample cell, and by measuring the time-dependent absorption spectrum in an initially solvent-filled part of the sample volume, we obtain the diffusion coefficients and UV/vis spectra of the species present in the sample solution. From these data, we construct a two-dimensional spectrum with absorption wavelength on one axis and diffusion coefficient (or equivalently, size) on the other, in which the UV/vis spectrum of a mixture with different molecular sizes is separated into the spectra of the different species, sorted by size. We demonstrate this method on mixed solutions of fluorescent dyes, biomolecules, and the UV-absorbing components of coffee, caffeine, and chlorogenic acid, all with concentrations in the μM range.

## Introduction

Ultraviolet and visible (UV/vis) absorption spectroscopy is a standard tool in most chemical and biochemical laboratories, with applications ranging from electronic-structure characterization^1−3^ to determining the concentrations of DNA and proteins, and characterizing aromatic compounds and tracking ligand binding.^4^ The UV and visible absorption wavelengths and intensities of a molecule provide information on its electronic structure, and additional solvatochromic and excitonic effects on the spectrum can be used as a probe of the conformation, as in the case of protein folding and DNA base pairing.^5−7^ Furthermore, UV/vis spectroscopy is nondestructive, comparatively inexpensive,^8^ and since UV/vis absorptions are quite strong, UV/vis spectroscopy can be used to analyze solutions with concentrations down to μM.

However, comparatively little information about the size of molecules or molecular assemblies can be obtained from the UV/vis spectrum, because electronic transitions generally occur in subunits of the molecules, such as the aromatic residues in proteins and the purine and pyrimidine bases in DNA and RNA. To obtain both size and UV/vis-spectral information, chromatographic methods are very well suited, and the combination of liquid chromatography and UV/vis detection can be used for e.g., biopolymer size characterization.^9^ However, these methods have limitations associated with the molecular size as well as the need for known calibrants to determine the relation between the retention behavior of the sample molecules and their size.^10^ Here, we present a different method to simultaneously obtain the size and UV/vis spectrum of molecules or particles in solution. Inspired by the elegant NMR concept of diffusion-ordered spectroscopy (DOSY),^11−23^ we demonstrate how a simple extension of a standard UV/vis spectrometer can be used to simultaneously characterize the electronic structure and the size of a molecule or particle. Similar to NMR-DOSY, the resulting two-dimensional spectra have absorption wavelength on one axis and diffusion coefficient on the other, and if there are several species with different sizes present in a solution, their individual spectra are separated and sorted by size. This method is an extension to visible and UV wavelengths of our previous work on infrared and Raman diffusion-ordered spectroscopy, of which the former was limited to infrared-transparent liquids.^24−26^

**Figure 1:**
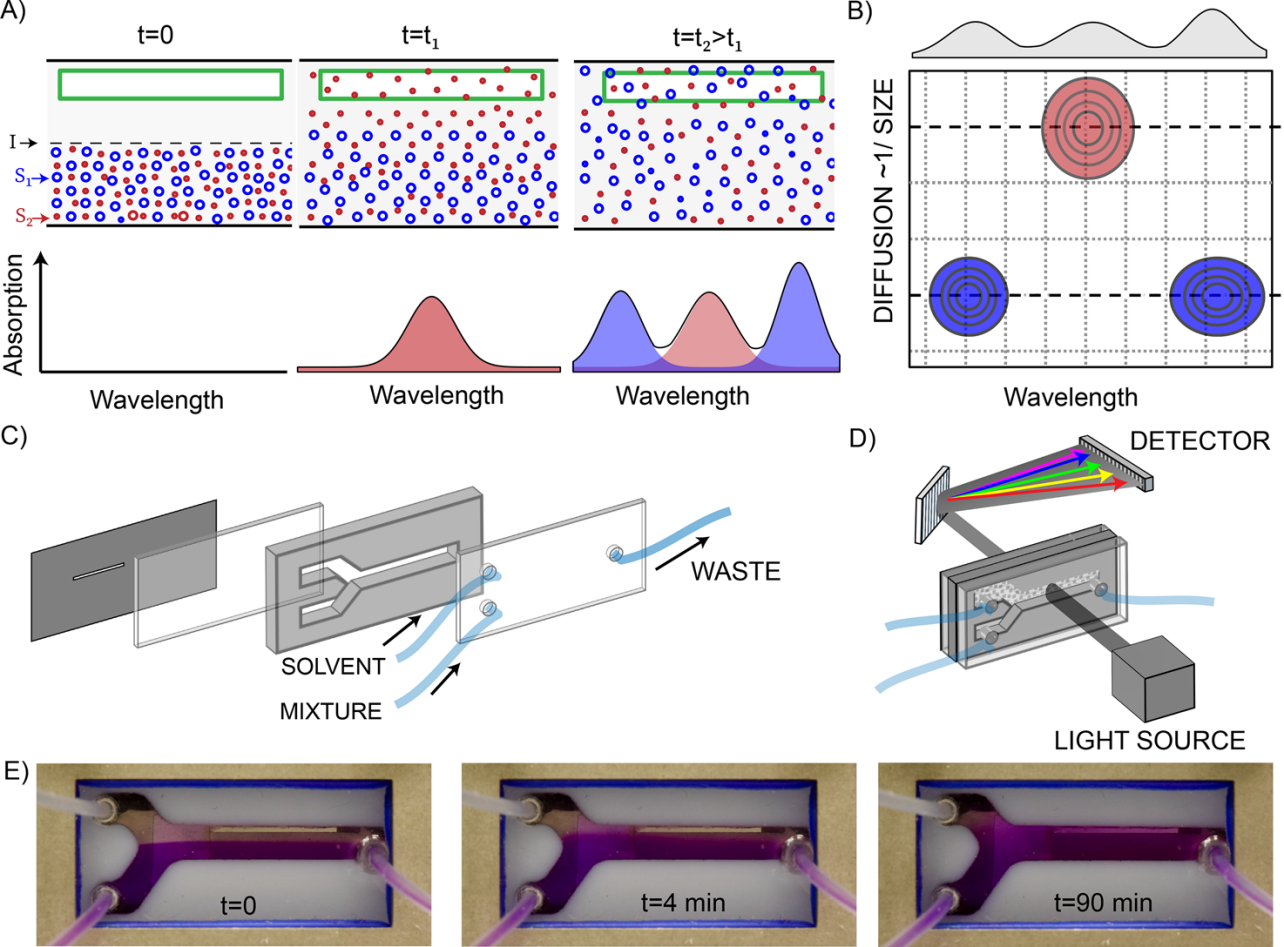
(A) Operation principle. At *t* = 0, we inject the sample solution in the bottom part of a transmission sample cell and solvent in the top part. Small molecules (red dots) diffuse faster than large ones (blue dots) into the initially solvent-filled part, so their absorption peaks appear first in the UV/vis spectrum recorded there. Spatial selectivity is achieved using a slit in the spectrometer beam (green rectangle). (B) Schematic two-dimensional DOSY spectrum, with absorption peaks ordered by the value of the diffusion coefficient of the molecules; (C,D) practical implementation. The size of the windows is 38.5 × 19 × 4 mm^3^. We use a UV/vis spectrometer with an array detector, but a scanning spectrometer can also be used. (E) Images of the channel at selected times after stopping the infusion of an aqueous solution of rhodamine and methylene blue in the bottom half of the channel, and water in the top half of the channel.

The method relies on the fact that the diffusion coefficient *D* of a molecule (or particle) is inversely proportional to its hydrodynamic radius *R* through the Stokes–Einstein relation.^27,28^ In Figure 1 we show how this effect can be used to simultaneously determine the sizes and UV/vis spectra of the species present in a solution. We simultaneously inject the sample solution and the pure solvent into a thin space between two UV-transparent windows, in such a way that the two liquids are in contact in a parallel flow. At *t* = 0, we stop the flow. The solute molecules (red and blue dots in Figure 1A) then diffuse from the solution-filled half into the solvent-filled half, each at a different rate determined by its diffusion coefficient.^29,30^ We measured the time-dependent UV/vis absorption spectrum at a position in the channel where there was initially only solvent. As time progresses, the solute molecules appear in the absorption spectrum at different rates depending on their size (in the figure: red first, blue later). The time dependence of the absorption amplitudes is given by a simple mathematical function (obtained by solving the diffusion equation), and a straightforward mathematical procedure transforms the time-dependence axis of the two-dimensional data set into a diffusion-constant (or equivalently, size-) axis (Figure 1B). In the following sections, we demonstrate how UV/vis-DOSY can be used to simultaneously obtain molecular sizes and electronic absorption spectra in three types of mixed solutions.

## Materials and Methods

### Sample Solutions

For the DOSY experiments, we prepared three mixed solutions in demineralized (milliQ) water: rhodamine B (chloride, Exciton Inc.) and methylene blue (Merck 52015); *N*-acetyl-l-tryptophanamide (Sigma-Aldrich, A6501) with ATP (disodium salt, Sigma-Aldrich A26-209) and lysozyme (Sigma-Aldrich, L4919); caffeine (Sigma-Aldrich, C0750) and chlorogenic acid (Sigma-Aldrich, C3878). To ensure laminar flow in the DOSY cell, we add 2.5 g/L polyethyleneglycol (4 M, Sigma-Aldrich) to the solutions. The solutions are degassed by placing them in an ultrasound bath for at least 15 min.

### Spectrometry Experiments

The sample solution and solvent are injected into the optical DOSY cell (Figure 1) at a rate of 0.1 mL/min using a Harvard PHD-ULTRA syringe pump. The cell consists of two UV-grade CaF_2_ windows (3 mm thick) separated by a 2.2 mm thick Teflon spacer. The sample solution and solvent are both injected into a 3 mm wide channel cut out in the Teflon spacer, each liquid filling up one-half of the sample volume. All measurements are performed using an HP/Agilent 8453 UV/vis spectrometer (equipped with a tungsten and a deuterium lamp, wavelength range 190–1100 nm, resolution 1 nm). Time series of spectra are obtained using a Labview script to automatically start spectral acquisition every minute. No wavelength scanning is required since the HP spectrometer is equipped with an array detector. The spectra are obtained as the average of three consecutive measurements, with 3 s of integration time per measurement. Between the measurements, the light beam in the spectrometer is blocked with an automated shutter to avoid temperature increase in the sample due to light absorption.

### Fit Function Used in the Data Analysis

To analyze the time-dependent spectral data and convert it into a DOSY spectrum, we solve the diffusion equation to obtain an expression for the time-dependent absorption (see the Supporting Information for the mathematical details). Sufficiently far away from the entrance and exit of the channel, the diffusion is effectively 1-dimensional. At *t* = 0, the spatial profile of the concentration is a step function, and the subsequent evolution of the position- and time-dependent concentration is described by the diffusion equation:
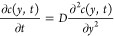
1with *D* the diffusion coefficient, and with initial concentration profile *c*(*y*,0) = 1 for −L/2 ≤ *y* < 0 and *c*(*y*,0) = 0 for 0 ≤ y ≤ L/2, where *L* is the width of the channel. Expressions in summation form for the complete solution are given in the Supporting Information. For our experiments, we are interested only in the concentration at the top edge of the channel, *c*(*L*/2,*t*). Introducing a dimensionless time variable τ = *Dt*/*L*^2^, we have *c*(*L*/2,*t*) = *CDt*/*L*^2^ = *C*(τ) with *C*(τ) as a function that depends only on the dimensionless parameter τ. Full summation expressions for *C* are given in the Supporting Information, but for practical applications, it suffices to use only the first few terms, and to obtain a relative precision of 10^–8^ (with respect to the exact solution) at all times, we can use
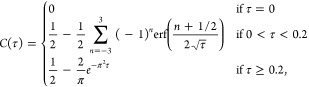
2where erf(*x*) is the Gauss error function. Note that *C* converges to , since after sufficient time the solute molecules will be distributed evenly over the two halves of the channel. The above equation is the concentration at *y* = *L*/2, whereas in the experiment, we use a slit with a finite width to spatially select the top part of the channel. Hence, we should actually integrate *c*(*y,t*) from *y* = *L*/2–*w* to *L*/2 (with *w* the slit width); but numerical calculation shows that if the slit is not too wide (*w* ≤ *L*/8) the integral can be approximated to within less than 1% by the concentration at *y* = *L*/2, so in the global fit of eq 3 explained below we use eq 2 instead of the more cumbersome integral.^24^

## Results and Discussion

### Proof-of-Principle Experiment with a Mixed Dye Solution

The practical implementation of optical DOSY is shown in Figure 1C–E. We use a liquid-sample cell suitable for transmission spectroscopy combined with a standard double syringe pump to inject the solution and solvent. The cell has two entrance holes and one exit hole, and we inject the sample solution into one entrance, and pure solvent into the other, at identical flow rates. After the pumping is stopped (at *t* = 0), the dissolved compounds diffuse into the solvent-filled half of the cell at a rate that depends on their diffusion coefficient and hence on their size. The cell is placed in a conventional UV/vis spectrometer to record the time-dependent absorption spectrum in the portion that is initially filled with solvent. The light beam in a standard UV/vis spectrometer typically has a diameter of several millimeters, which is too large to selectively sample the relevant part of the sample volume. To ensure that we measure the absorption spectrum of a specific region of the volume, we mount a optical slit onto the sample cell (indicated by the green rectangle in Figure 1A).

**Figure 2:**
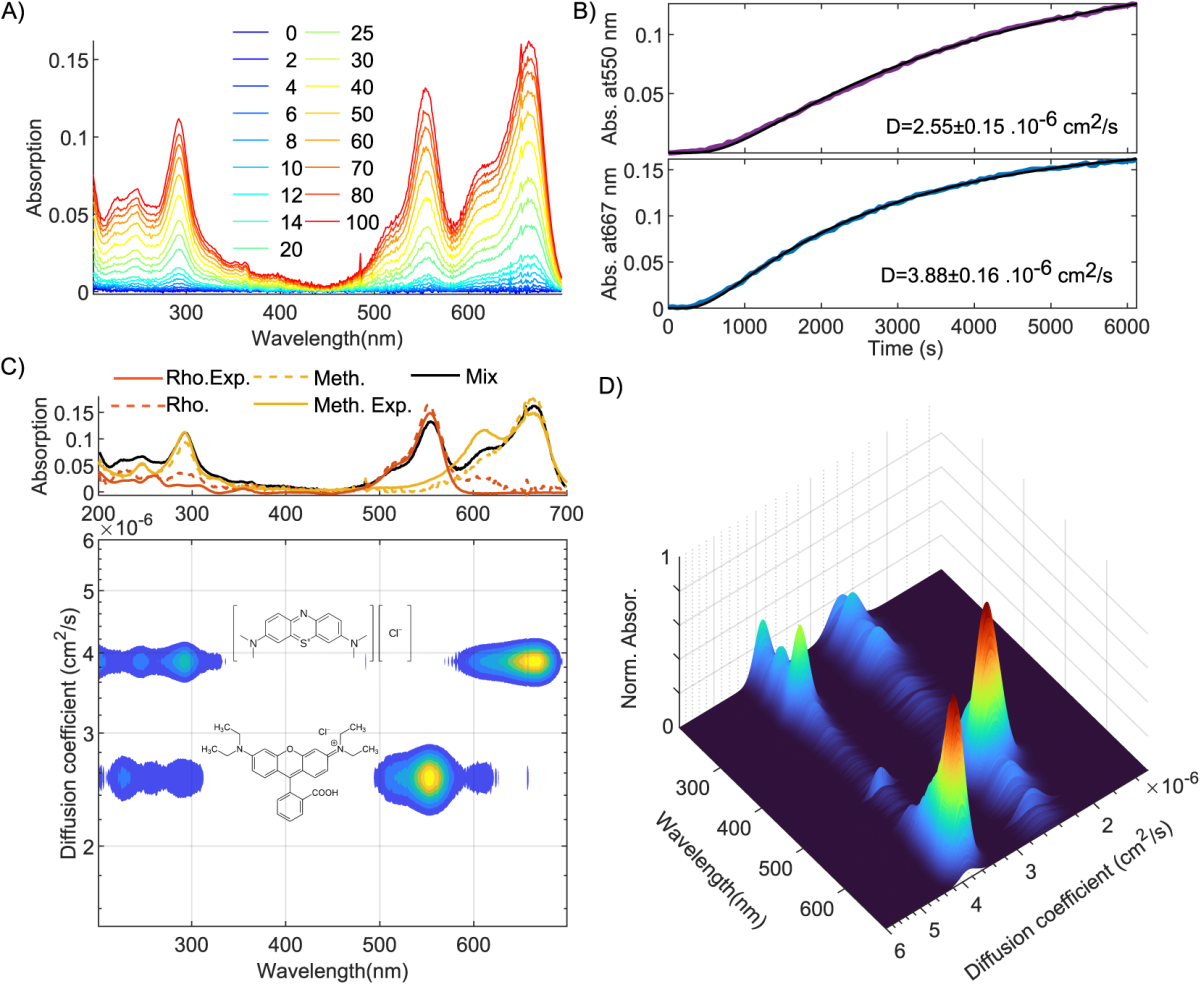
UV/vis-DOSY on a mixed solution of rhodamine B and methylene blue (water background absorption subtracted). (A) UV/vis absorption spectra at selected times after injection of the mixed solution (in minutes); (B) time-dependent absorption at 550 nm (absorption maximum of rhodamine B) and 667 nm (absorption maximum of methylene blue). The black curve is the result of a least-squares fit, with the diffusion coefficient as the fit parameter; (C) a contour plot of the UV/vis-DOSY spectrum obtained from a sequential fit to the data of (A), showing which peaks in the absorption spectrum of the mixture (shown in the top panel) belongs to which molecule. Top panel: DOSY-extracted (dotted curves) and separately measured spectra (continuous curves) of the two components. (D) Surface plot of the UV/vis-DOSY spectrum.

To test the method, we start with a solution of two dye molecules, rhodamine B and methylene blue (chemical structures shown in Figure 2C), which have distinctly different UV/vis absorption spectra, with maxima at 555 and 665 nm respectively. The spectrum of a mixture of these dyes (11 μM rhodamine B, 30 μM methylene blue) is shown in the top panel of Figure 2C. We inject the mixed solution into the lower part of the channel and water into the top part, stop the flow at *t* = 0 and then record consecutive UV/vis spectra at the top of the water-filled part. The result is shown in Figure 2A. Initially, the absorption is zero everywhere, but with increasing time, the absorption spectra of rhodamine B and methylene blue appear as these molecules diffuse into the water-filled part of the cell. The methylene blue peak (555 nm) appears faster than the rhodamine B peak (665 nm); see Figure 2B. This difference is due to the lower diffusion coefficient of rhodamine B compared to methylene blue, which is a consequence of the larger size of the rhodamine B molecules. A size difference translates directly into a difference in diffusion coefficient *D* through the Stokes–Einstein equation: , where *k*_B_ is Boltzmann’s constant, *T* the temperature, η the viscosity of the solvent and *R* the hydrodynamic radius of the diffusing molecule.^27,28^ Thus the time dependence of the UV/vis peaks, which is governed by the diffusion coefficient of the absorbing molecules, mirrors their diffusion coefficients and, hence, their size.

The time-dependent spectral data of Figure 2A can be converted to a two-dimensional spectrum that has wavelength on one axis and diffusion coefficient on the other. To obtain such a DOSY spectrum from the time- and frequency-resolved data, we quantitatively analyze the data using the diffusion equation. It is easily derived that the time-dependence of the concentration at the top of the channel is determined only by the channel height and the diffusion coefficient; and by solving the diffusion equation (see Materials and Methods section) one finds that the normalized time-dependent concentration at that position is given by a universal function *C*(*Dt*/*L*^2^) where *t* is time, *D* the diffusion coefficient, *L* the channel height (see eq 2 above for the explicit expression of this function *C*). If there are *N* species present in the solution, with absorption spectra *A*_*i*_(λ) (with *i* the species number and λ the wavelength), then the total absorption at wavelength λ and time *t* is given by
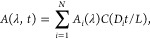
3where *N* is the number of species in the mixture (in this case 2), *D*_*i*_ is the diffusion coefficient of species *i*, and *L* is the channel width. Since the two dyes have distinct absorption peaks, we can determine their diffusion coefficients in a straightforward manner, by least-squares fitting the expression for the time-dependent absorption of a single compound (eq 2) to the time-dependent absorptions at 550 and 667 nm. In this way we obtain accurate estimates for *D*_1_ and *D*_2_, and subsequently fitting eq 3 (with *N* = 2) to the entire time- and wavelength dependent data set (keeping *D*_1_, *D*_2_ fixed), we obtain the spectra *A*_*i*_(λ) associated with the two diffusion coefficients.

From this combined data, we obtain the optical-DOSY spectrum *S*(λ, *D*) by multiplying the spectral amplitude *A*_*i*_(λ) with the appropriate probability distribution for *D*_*i*_:^15^
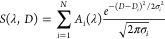
4where σ_*i*_ are the uncertainties in the diffusion coefficients as obtained from the global least-squares fit. Note that this 2D-DOSY spectrum is essentially a stack of 1D spectra (one for each *D*_*i*_ value).^11,12^

Figure 2C,D shows the UV/vis-DOSY spectrum of the two-component dye solution obtained in this way. In the DOSY spectrum, the total absorption spectrum (shown in the top panel of Figure 2C) is separated into two sets of peaks at different *y*-coordinates (=diffusion coefficients), each corresponding to one of the dyes present in the solution, and the value of the diffusion coefficient provides information about the size. Our experimental values for the diffusion coefficients are lower (about 35%) than the previously reported values,^31,32^ probably due to the presence of PEG (polyethylene glycol), which we use to ensure laminar flow.^33^ Since rhodamine B in aqueous solutions occurs as a mixture of zwitterionic and cationic forms,^34^ and the exchange between these species occurs much faster than the time scale of our experiment, the diffusion coefficient observed for rhodamine B is the average of those of the zwitterionic and cationic forms.

In the case of unknown molecules in a mixed solution, the value of the diffusion coefficient can be used to obtain an estimate of the size of the compounds, and the UV/vis spectrum associated with each diffusion constant provides information on the electronic structure of the molecule or particle. The top panel of Figure 2C shows the spectra obtained from the data analysis, together with separately measured absorption spectra of the two dyes. Around 600 nm there is some “cross talk”, but overall, there is good agreement between the DOSY-extracted and separately measured spectra. The small differences are probably due to our data analysis procedure, which we are still optimizing.

If the extinction coefficients of the components are known, then the *A*_*i*_(λ) spectra (horizontal slices of the DOSY spectrum) can be used to determine their concentrations in the sample solution. In this example, the spectrum contains isolated peaks for each component (so that the diffusion coefficient can be determined from analyzing the time-dependence at the corresponding wavelengths), but this is not a necessary requirement, as we will see next.

### Application to Mixed Caffeine and Chlorogenic Acid Solution

**Figure 3:**
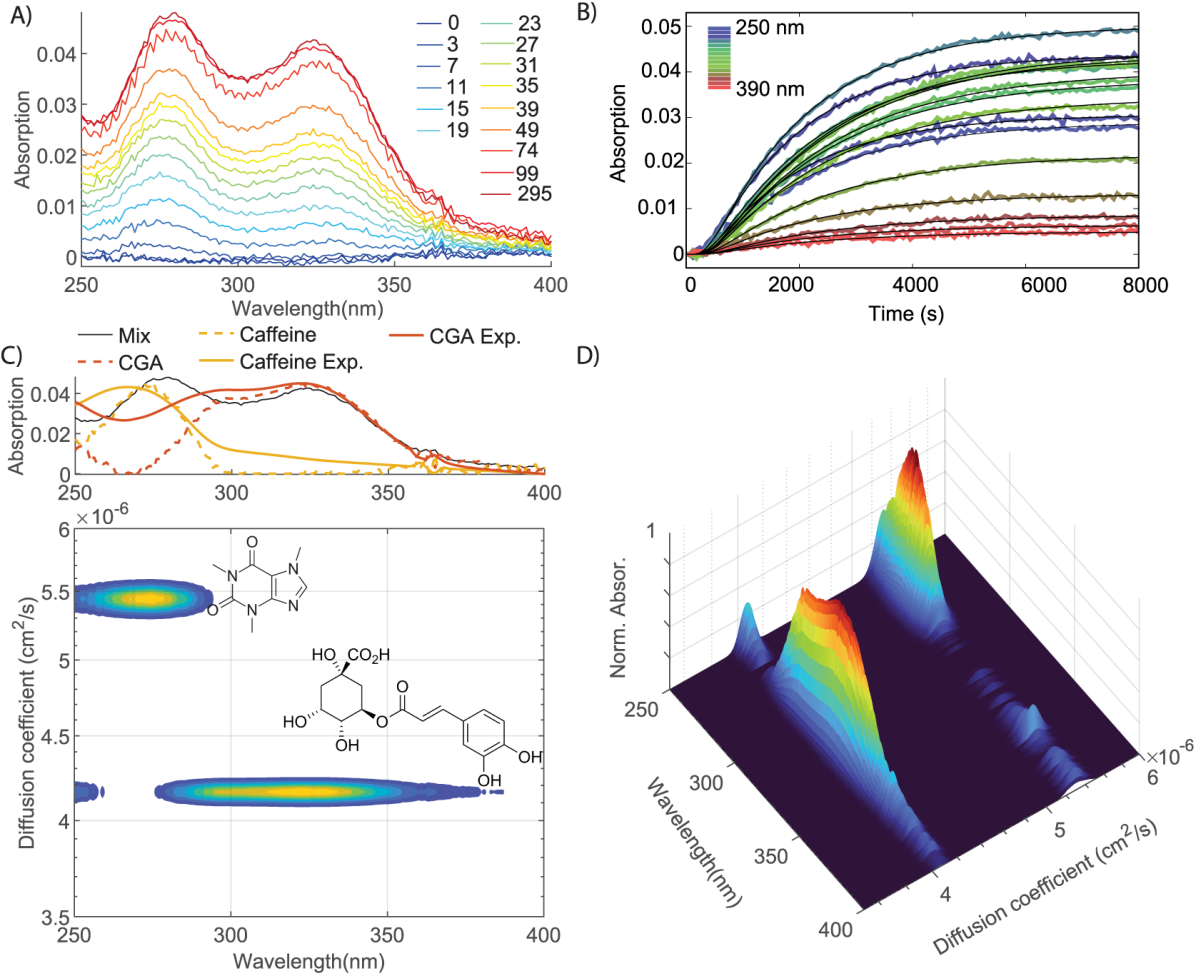
UV/vis-DOSY of a mixed solution of 25 μM caffeine and 25 μM chlorogenic acid (CGA). (A) UV-absorption spectra at selected times (in minutes) after injection of the mixed solution; (B) time-dependent absorption for selected wavelengths (separated by 10 nm). In each plot, the black curves are the results of the global fit performed through the whole data set; (C) Top panel: UV absorption spectrum of the solution and the DOSY-extracted (dotted curves) and separately measured (continuous curves) spectra of the two components. Bottom panel: contour plot of the UV/vis-DOSY spectrum obtained from the a global fit to the data of (A), showing which peaks in the UV/vis spectrum of the mixture (shown in the top panel) belong to which molecule; (D) surface plot of the UV-DOSY spectrum.

We now turn from visible to UV wavelengths, as most organic and biomolecules do not absorb at visible wavelengths but only in the UV. To illustrate a potential practical application of UV/vis-DOSY, we use it to disentangle the UV absorption spectrum of a mixed solution containing two important components of coffee: caffeine and chlorogenic acid (CGA). Knowing the concentrations of these two compounds in coffee is important for various purposes, such as assessing coffee quality and investigating its impact on consumer health,^35,36^ and several analytical methods are commonly used to quantify them.^37−41^ A particularly simple method for determining the concentrations of caffeine and CGA in coffee is UV absorption spectrometry,^42−44^ which makes use of the fact that caffeine and CGA are the main two compounds in brewed coffee that absorb in the UV. However, their UV absorption bands are broad and overlap strongly, which complicates the analysis of the UV spectrum. Since caffeine and CGA have different sizes (see Figure 3 for their chemical structures), with UV/vis-DOSY the individual contributions of caffeine and CGA to the UV absorption spectrum can be easily separated.

We demonstrate this idea on a mixed solution containing 25 μM caffeine and 25 μM CGA. Figure 3A shows the time-dependent UV spectrum recorded in a UV-DOSY measurement. The band around 275 nm is due to caffeine, the bands around 295 and 325 nm to CGA.^42,43^Figure 3B shows the time-dependent absorption in a UV-DOSY experiment at selected wavelengths. Due to the overlap of the species spectra, we cannot determine the individual diffusion coefficients by analyzing selected wavelengths as we did for the mixed dye solution above. To convert the wavelength- and time-dependent absorption *A*(λ,*t*) into a DOSY spectrum, we perform a global least-squares fit of eq 3 to the two-dimensional data set, where the diffusion coefficients *D*_*i*_ and the spectra *A*_*i*_(λ) of the two species are treated as fit parameters (note that the number of data points is much larger than the number of free parameters by a factor of approximately the number of time points, so the fit is well defined). From the least-squares fit (shown at selected wavelengths as the curves in Figure 3B) we obtain the diffusion coefficients, *D*_*i*_, and the associated absorption spectra, *A*_*i*_(λ), of each species. The root-mean-square deviation between fit and data is 0.5 mOD, which is comparable to the noise level in the measurements. The small deviations between the fit and the experimental data might be due to caffeine and CGA dimers in our sample (although this should be a small effect, since from the dimerization–equilibrium constants^45^ we estimate dimer fractions of below 2.5% for CGA and below 1.4% for caffeine at our sample concentrations).

Figure 3C,D shows the UV/vis-DOSY spectrum obtained from the global fit of eq 3 to the full-time- and frequency-dependent data set. The peaks are distributed in two rows located at the diffusion coefficient values corresponding to the two different molecular sizes present in the solution. In the bottom row, we observe two signatures at 295 and 325 nm due to CGA, and in the top row, the peak at 275 nm due to caffeine. The observed diffusion coefficients for caffeine and CGA are 5.44 ± 0.08 × 10^–6^ and 4.16 ± 0.05 × 10^–6^ cm^2^/s, respectively, the difference being as expected from the different sizes of caffeine and CGA. For caffeine the diffusion coefficient has been reported previously, and our value corresponds to ∼ 70% of the literature value,^46^ the slower diffusion probably being due to the presence of added PEG in our solution. This example demonstrates that UV-DOSY can spectrally separate compounds with relatively small differences in diffusion coefficient (in this case, about 20%). The spectrum of CGA obtained from the DOSY experiment nicely matches the separately measured spectrum. The DOSY-extracted spectrum of caffeine is somewhat narrower than the separately measured spectrum. As in the case of the mixed dye solution, we believe that this small mismatch is due to our data-analysis procedure.

### Application to a Mixed Solution of Biomolecules

For many biomolecules in solution, measuring the UV absorption is a convenient way to determine their concentrations, and in some cases also to obtain some information on the electronic structure of the molecule.^5−7^ In particular, the UV absorbance of proteins (which have a characteristic absorption band at ∼280 nm due to tyrosine and tryptophan residues, and at ∼260 nm due to phenylalanine residues)^5^ is commonly used to estimate protein and DNA concentrations.^4^ Extending this idea to UV-DOSY, we can simultaneously obtain the size (hydrodynamic radius) and UV absorption spectrum of a biomolecule. Moreover, we can also use UV-DOSY to study mixed solutions, resulting in a separation of the spectrum according to the molecular size. This is particularly useful because UV absorption bands are often broad and overlapping, which limits the usefulness of conventional UV absorption spectroscopy to characterize mixed solutions of biomolecules. In the optical-DOSY spectrum, the absorption spectra of the components in the mixture are sorted by their diffusion coefficient (or equivalently, size).

**Figure 4:**
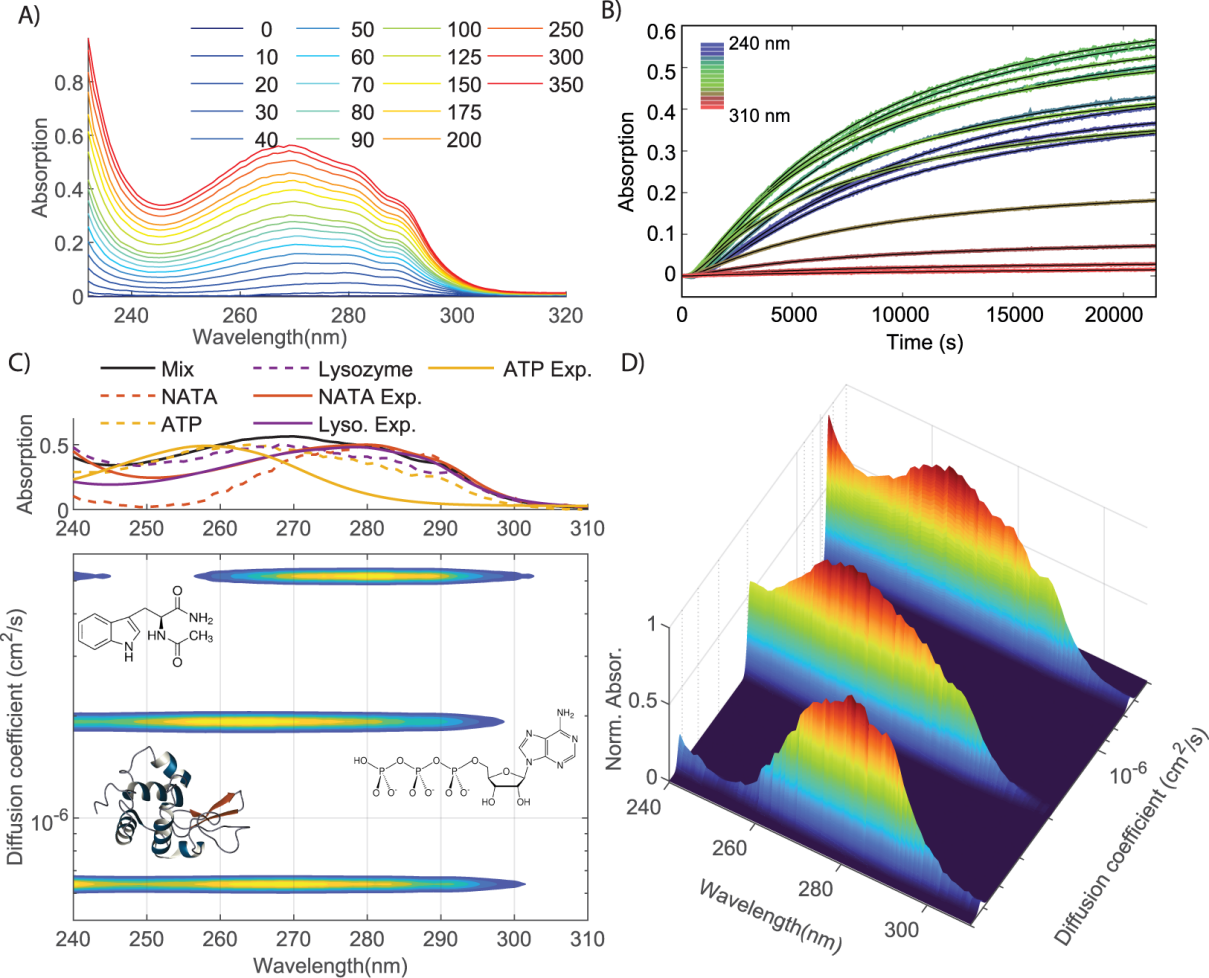
UV/vis-DOSY on a mixed solution of *N*-acetyl-tryptophan-amide (NATA), ATP, and lysozyme. (A) UV-absorption spectra at selected times after injection of the mixed solution (in minutes); (B) time-dependent absorption for selected wavelengths (separated by 5 nm). In each plot, the black curves are the results of the global fit performed through the whole data set. (C) Top panel: UV absorption spectrum of the mixed solution and DOSY-extracted (dotted curves) and separately measured (continuous curves) spectra of the three components. Bottom panel: contour plot of the UV/vis-DOSY spectrum obtained from the global fit to the data of (A), showing which peaks in the UV/vis spectrum of the mixture (shown in the top panel) belong to which molecule; (D) surface plot of the UV/vis-DOSY spectrum.

Figure 4 shows an example of a mixed solution containing lysozyme, ATP, and the small peptide *N*-acetyl-tryptophan-amide (NATA). The spectrum of this mixture exhibits a broad blob in the 250–300 nm region (Figure 4C, top panel). ATP has an absorption maximum at ∼260 nm,^5^ whereas lysozyme and NATA both have a broad UV band at ∼280 nm (due to Trp, Tyr, and Phe residues absorbing at ∼280, 275, and 260 nm, respectively).^5^ These bands overlap so strongly that the ATP peak cannot even be distinguished in the conventional UV absorption spectrum. In the time-dependent absorption spectrum (Figure 4A), the absorption at 280 nm rises faster than that at 260 nm due to the faster diffusion of NATA (Figure 4B). From a global least-squares fit of eq 3 with *N* = 3, and using a least-squares fit of eq 4 to the data (rms deviation = 1.0 mOD), we obtain the UV-DOSY spectrum shown in Figure 4C,D. In this spectrum the three contributions to the UV absorption are separated by size, and the spectral overlap problem has been solved: the spectra of the three molecules present in the solution are separated by size, and separately accessible. In the top panel, we compare the DOSY-extracted spectra to separately measured component spectra. Although the bands in the DOSY-extracted spectra are somewhat broader than those in the separately measured component spectra, the central wavelengths of the bands in the spectra match nicely.

### Practical Aspects

The above experiments show that optical DOSY can be a useful method to simultaneously characterize molecular size and electronic structure obtained from the diffusion constant and the UV/vis absorption spectrum. We now discuss a number of practical aspects.

#### Determining the Number of Components Required to Analyze Mixed Solutions

In the experiments shown here, the number of components needed in the global-fit analysis of the data was known, but in practice, this might not always be the case. In the case of a sample with an unknown number *N* of components, this number (provided that it is not too large) can be determined from a singular-value decomposition^47,48^ (or any other decomposition strategy, e.g., principal component analysis) of the two-dimensional *A*(λ,*t*) data set. By eq 3, the data is a sum of *N* products of λ and *t*-dependent left- and right-singular vectors.^47^ In fact, a singular-value decomposition of the data of Figure 2A shows two significant components (all components with smaller weights containing noise), and a singular-value decomposition of the data of Figure 4A shows three (see SI). Thus, the number of species to be used in analyzing the data (*N* in eq 3) can be determined from the data itself. It should be noted that the number of components obtained in this way provides a lower bound for the number of chemical species in the sample because if two species have very similar or identical diffusion coefficients, they will give rise to a single singular vector in the singular-value decomposition of the time-dependent data. A next step in the case of an unknown number of components could be to perform a least-squares fit of eq 2 to the time-dependent absorption at the wavelengths where the absorption spectrum of the sample has a maximum. For highly complex systems an iterative deflation^49,50^ of the components/singular values can then provide an accurate estimation of the true number of components. Moreover, methods such as multivariate curve resolution, applied iteratively can also be used to resolve complex systems.

#### Continuous Size Distributions

An analysis in terms of a discrete set of sizes and spectra will not work in the case of polydisperse mixtures with continuous size distributions, such as polymer or nanoparticle solutions, or in the case of diffusion coefficients with overlapping uncertainties. Fortunately, sophisticated data analysis methods have been developed for NMR-DOSY on such samples,^51−54^ and we think that this theoretical framework can be adapted without too much difficulty to analyze optical DOSY data.

#### Large Molecules/Particles

A different issue arises if the molecular or particle size is large (≫10 nm). In that case, the diffusion coefficient is so low that diffusion over millimeter distances would take prohibitively long. This can be remedied in two ways. If a mixed sample contains only one single very large species, then a simple approach is to measure the time-dependent absorption spectrum in the solution-filled half of the channel, thus tracking the depletion of all the other, smaller solute molecules due to their diffusion. In this way all the species spectra can obtained, as well as the diffusion coefficients except the one of the largest species.^26^ A more generally applicable method for studying larger compounds is to reduce the distance over which they diffuse in the experiment. The characteristic time for the diffusion to spread a molecule over the entire channel width is . This means that using a narrower channel can decrease the waiting time, and using a 10 times narrower channel reduces the measurement time by a factor of 100. With such a narrower channel, spatial selection of the optical probing can no longer be done with a slit because the transmission would be too low (leading to unworkable signal-to-noise levels). However, inserting a small (Kepler-type) telescope with the focus at the sample position into the UV/vis spectrometer solves this problem, and we are currently implementing this. The use of a narrower channel also reduces the total measurement time, which is useful in the case of photosensitive compounds.

#### Sample Concentrations

The extinction coefficients of molecules containing one or more double bonds is typically on the order of 10^4^ cm^–1^ M^–1^,^4^ and absorptions of 0.05 OD (and even less) can easily be detected, so concentrations on the order of 5 μM are sufficient for an optical-DOSY measurement. By using thicker sample cells, this number can be decreased if necessary.

#### Comparison with Chromatography Methods and NMR-DOSY

An estimate of molecular sizes can also be obtained by chromatographic methods. The chromatographic method that seems most similar to UV/vis-DOSY is hydrodynamic chromatography, which is somewhat more difficult to operate than UV/vis-DOSY, generally requires calibrants, and cannot so easily handle higher sample dimensionality (i.e., chemicals from different families). Taylor-dispersion analysis (TDA) is also commonly used for estimating the size of particles and molecules in solution,^55^ but mixed samples are not so easy to analyze with this method,^56,57^ and to obtain information on the electronic structure of the molecules coupling to a dedicated spectrometer is necessary. Hence, we think that UV/vis-DOSY can be a useful complement to these chromatographic methods.

Compared to NMR spectra, UV/vis spectra contain far less structural information, and this also holds for optical DOSY. However, there might be situations where the NMR spectra of species in a mixed solution overlap strongly, but the UV/vis spectra are different: In this case, optical DOSY might be a useful addition to NMR-DOSY. Furthermore, the UV/vis experiments do not require deuterated solvents and can be done on paramagnetic compounds (which are not accessible to NMR), and the experiments can be done at comparatively low concentrations (see above). Furthermore, using nonlinear multidimensional spectroscopy^58−61^ it becomes possible to obtain more structural information from the UV/vis spectra of molecules, and combining DOSY with such nonlinear optical spectroscopy should be comparatively easy to do.

## Conclusion

In summary, we present a simple and inexpensive method to simultaneously measure the size and characterize the electronic structure of molecules and particles in solution by providing their UV/vis absorption spectrum together with their diffusion coefficient. The method can be used to characterize solutions containing a single compound but also to investigate mixed solutions, giving two-dimensional spectra in which the UV/vis spectra of species with different sizes appear at different positions on the diffusion-coefficient axis, similar to the two-dimensional spectra obtained from NMR-DOSY, but with optical wavelength rather than chemical shift on the horizontal axis. As the size separation relies on the diffusion coefficient, we believe that a size resolution similar to that of NMR-DOSY should be achievable, and we hope to achieve this goal by relying on the data-analysis algorithms that have already been developed for NMR-DOSY.

## Supporting Information Available

Details of the data analysis; singular-value decomposition (PDF)
