# Supplementary material for: UV/Visible Diffusion-Ordered Spectroscopy: A Simultaneous Probe of Molecular Size and Electronic Absorption
Source: Anal Chem. 2024 Sep 10;96(38):15168–76. doi: 10.1021/acs.analchem.4c02026 (PMC11428122; doi:10.1021/acs.analchem.4c02026)
Supplement: Supplementary file 1 — ac4c02026_si_001.pdf [file ac4c02026_si_001.pdf]

SUPPORTING INFORMATION

UV/Visible Diffusion-Ordered Spectroscopy: A Simultaneous Probe of Molecular Size and Electronic Absorption

Giulia Giubertoni,* Marina Gomes Rachid, Carolyn Moll, Michiel Hilbers, Saer Samanipour, and Sander Woutersen*

Van 't Hoff Institute for Molecular Sciences, University of Amsterdam, Science Park 904, 1098XH Amsterdam, The Netherlands

E-mail: g.giubertoni@uva.nl; s.woutersen@uva.nl

Details of the data analysis

Time- and position-dependent absorption

To analyze the time- and wavelength-dependent absorption, and obtain the diffusion coefficients and their associated spectra from the data, we solve the diffusion equation and least-squares fit the solution to the experimental data matrix. Sufficiently far away from the entrance and exit of the channel (see schematic below), the diffusion is effectively 1-dimensional (i.e., the concentration is independent of x). At $t = 0$, the concentration profile of each species is a step function, and the

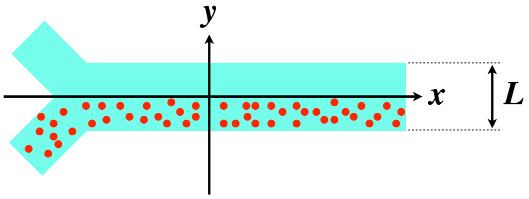

subsequent evolution of the position- and time-dependent concentrations is described by the diffusion equation (with different diffusion coefficients for different species). For a channel of width L centered at $y = 0$ (see schematic), the diffusion equation for the time and position dependent concentration $c(y,t)$ of a particular species

$$\frac{\partial^2 c(y,t)}{\partial y^2} = D\frac{\partial c(y,t)}{\partial t} \tag{1}$$

with D the diffusion coefficient, and with initial concentration profile $c(y,0) = 1$ for $-L/2 \leq y < 0$ and $c(y,0) = 0$ for $0 \leq y \leq L/2$, and boundary condition $\partial c/\partial y = 0$ for $y = \pm L/2$ (no flux at the boundaries). Introducing a dimensionless length $\xi = y/L$ and a dimensionless time $\tau = Dt/L^2$, the solution of the equation is $c(y,t) = C(y/L, Dt/L^2)$, where[1]

$$C(\xi,\tau) = \frac{1}{2} - \frac{2}{\pi}\sum_{n=0}^{\infty}\frac{\sin\left[\pi(2n+1)\xi\right]e^{-\pi^2(2n+1)^2\tau}}{2n+1}. \tag{2}$$

For $\tau \ll 1$ this sum converges slowly, and then it is more efficient to use the image-charge method[2]

which gives

$$C(\xi,\tau) = \frac{1}{2} - \frac{1}{2}\sum_{n=-\infty}^{\infty} \mathrm{erf}\left(\frac{\xi+n}{2\sqrt{\tau}}\right), \tag{3}$$

where $\mathrm{erf}(x) = (2/\sqrt{\pi})\int_0^x e^{-t^2}dt$. To efficiently calculate $c(y,t)$, we use eq. (2) for $\tau > 0.2$ and eq. (3) for $\tau < 0.2$. Using the first two terms of eq. (2), and the terms $-3 \leq n \leq 3$ of eq. (3), we obtain a precision at $x = L/2$ of $< 10^{-8}$ all times. The expression describes the concentration (and hence absorption) profile of a single component (see e.g. Fig. 2B in the main text). When there are N species in the solution, with UV/Vis-spectra $A_i(\lambda)$ (with i the species number and λ the wavelength), the total absorption at wavelength λ, position y and time t is given by

$$A(\lambda,y,t) = \sum_{i=1}^{N} A_i(\lambda)C(y/L, D_i t/L^2), \tag{4}$$

where D_i is the diffusion coefficient of species i. In the UV/Vis-DOSY experiments, we measure the UV-Vis absorption in a spatial range close to the edge of the channel, selected using a slit. In this case we should integrate eq. (4) from $y = L/2 - w_0$ to $L/2$ (with w_0 the slit width), but numerical calculation shows that if the slit is not too wide (w_0 less than $\frac{1}{8}$ of the channel width L), the integral can be approximated to within less than 1% by the concentration at $y = L/2$, and so we can fit the data by

$$A(\lambda,L/2,t) = \sum_{i=1}^{N} A_i(\lambda)C(1/2, D_i t/L^2). \tag{5}$$

Obtaining the UV/Vis-DOSY spectrum from the data

To obtain DOSY spectra from the time- and frequency-resolved data, we use a procedure similar to equation (2) of Ref. 3. By least-squares fitting eq. 5 to the data, we obtain diffusion constants D_i and their associated spectra $A_i(\lambda)$. The UV/Vis-DOSY spectrum $S(\lambda,D)$ is obtained by multiplying the spectral amplitude $A_i(\lambda)$ with the appropriate probability distribution for D_i:[3]

$$S(\lambda,D) = \sum_{i=1}^{N} A_i(\lambda)\frac{e^{-(D-D_i)/2\sigma_i^2}}{\sqrt{2\pi\sigma_i}}$$

where N is the number of species ($N = 2$ in Fig. 3, $N = 3$ in Fig. 4), and σ_i are the uncertainties in

the diffusion coefficients obtained from the least-squares fits.

Singular-value decomposition

As discussed in the main text, in the case of a mixed sample in which the components have strong spectral overlap, the number of components needed in the global least-squares fit can be estimated from a singular-value decomposition. In Figures S1 and S2 we show the singular-value decompositions of the the cafeine/CGA and NATA/ATP/BSA data sets discussed in the main text. In each case, the number of physically meaningful vectors equals the number of components in the sample, and the remaining vectors contain noise contributions to the signal.

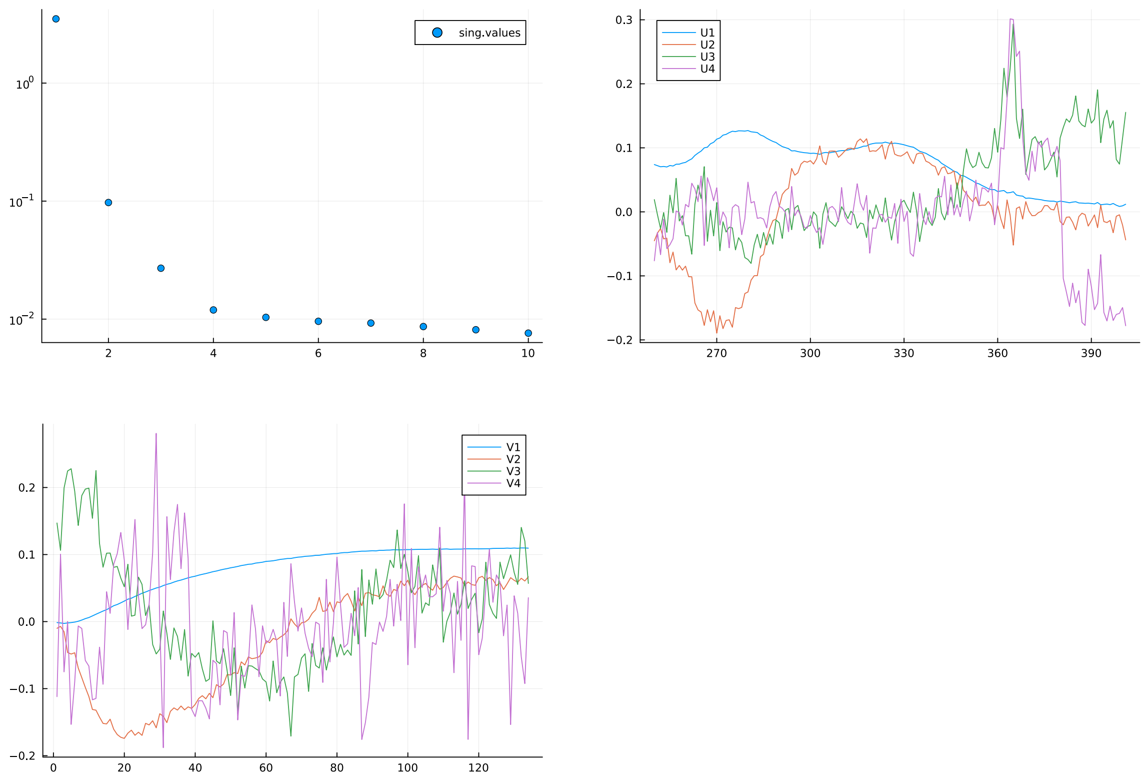

Figure S1: Singular-value decomposition of the cafeine/CGA data (Figure 3 of the main text). Left-top panel: first 10 weights; right-top panel: spectral singular vectors of the first 4 components; lower panel: first 4 time-dependent singular vectors. Only the first two spectral vectors contain physically meaningful data, the other vectors contain noise contributions.

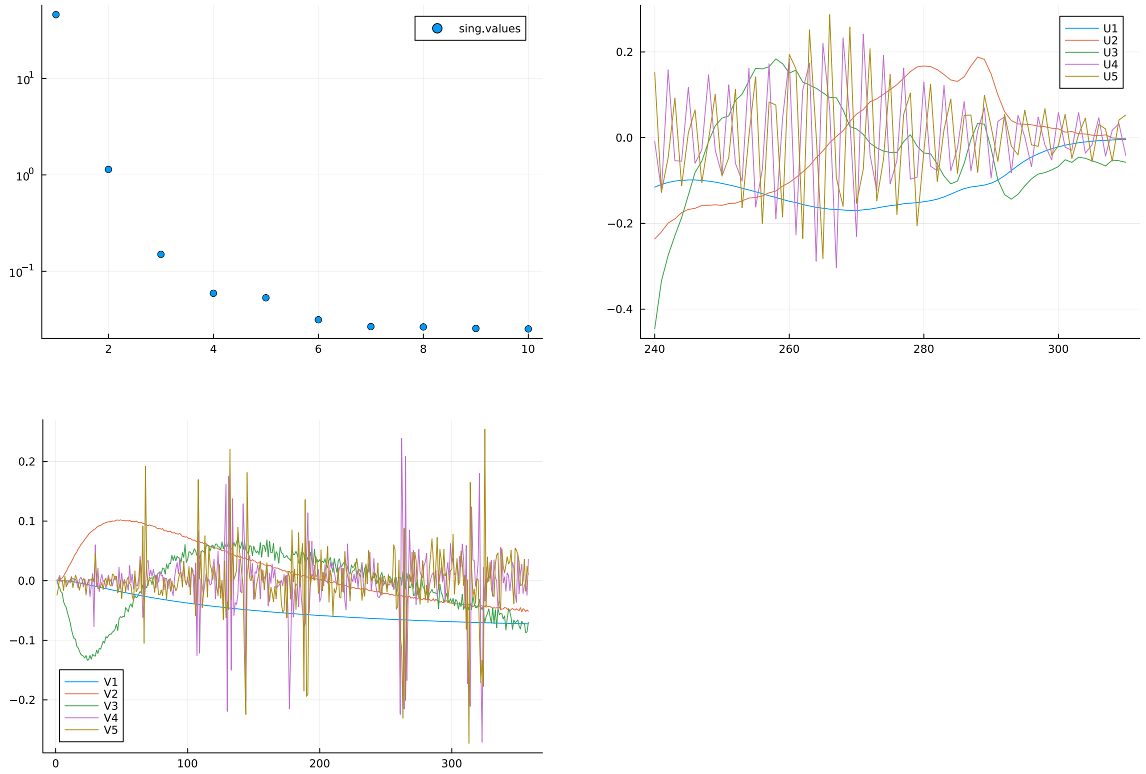

Figure S2: Singular-value decomposition of the NATA/ATP/BSA data (Figure 4 of the main text). Left-top panel: first 10 weights; right-top panel: spectral singular vectors of the first 5 components; lower panel: first 5 time-dependent singular vectors. Only the first three spectral vectors contain physically meaningful data, the other vectors contain noise contributions.

References

(1) Ghez, R. *A Primer of Diffusion Problems*; Wiley: New York, 1988.

(2) Balakrishnan, V. *Elements of Nonequilibrium Statistical Mechanics*; Springer, 2021; pp 97–106.

(3) Morris, G. A. In *Encyclopedia of Magnetic Resonance*; Harris, R. K., Wasylishen, R. E., Eds.; Wiley: Chichester, UK, 2009.
